# Isolation, Separation, and Preconcentration of Biologically Active Compounds from Plant Matrices by Extraction Techniques

**DOI:** 10.1007/s10337-017-3405-0

**Published:** 2017-09-22

**Authors:** Victoria Raks, Hossam Al-Suod, Bogusław Buszewski

**Affiliations:** 1 Interdisciplinary Centre of Modern Technologies, Nicolaus Copernicus University, 4 Wileńska Str., 87-100 Toruń, Poland; 2 Chair of Environmental Chemistry and Bioanalytics, Faculty of Chemistry, Nicolaus Copernicus University, 7 Gagarina Str., 87-100 Toruń, Poland; 3 Department of Analytical Chemistry, Faculty of Chemistry, Taras Shevchenko National University of Kyiv, Volodymyrska Street, 64/13, Kyiv, 01601 Ukraine

**Keywords:** Biologically active compounds, Estrogenic properties, Polyols, Flavonoids, Oligosaccharides, Solid-phase extraction, Membrane extraction

## Abstract

Development of efficient methods for isolation and separation of biologically active compounds remains an important challenge for researchers. Designing systems such as organomineral composite materials that allow extraction of a wide range of biologically active compounds, acting as broad-utility solid-phase extraction agents, remains an important and necessary task. Selective sorbents can be easily used for highly selective and reliable extraction of specific components present in complex matrices. Herein, state-of-the-art approaches for selective isolation, preconcentration, and separation of biologically active compounds from a range of matrices are discussed. Primary focus is given to novel extraction methods for some biologically active compounds including cyclic polyols, flavonoids, and oligosaccharides from plants. In addition, application of silica-, carbon-, and polymer-based solid-phase extraction adsorbents and membrane extraction for selective separation of these compounds is discussed. Potential separation process interactions are recommended; their understanding is of utmost importance for the creation of optimal conditions to extract biologically active compounds including those with estrogenic properties.

## Introduction

Free radicals can cause damage in the human body when they interact with important cellular components such as DNA, or the cell membrane, making the body susceptible to many diseases such as atherosclerosis, diabetes, rheumatism, inflammation, age-related eye disease, and cancer [1, 2]. To prevent such harmful effects of free radicals, it is recommended to maintain a healthy diet, rich in phytochemicals such as polyphenols, flavonoids, oligosaccharides, and cyclic polyols including cyclitols (inositols) [3–5]. Most of these compounds are widely present in various fruits, herbs, and honey.

Attempts to isolate cyclic polyols from natural sources are considered highly valuable technical aims due to their wide range of applications in pharmaceutical manufacturing [6–8]. Altered levels of polyols have been observed in certain neurological disorders leading to diabetic neuropathy. This type of diabetes is associated with elevated levels of sorbitol and reduced levels of *myo*-inositol in neuronal tissues including cerebrospinal fluid [9]. *myo*-Inositol is classified as a member of the vitamin B complex (vitamin B8), produced from glucose-6-phosphate in humans by a highly conserved process known as the Loewus pathway [10]. Several studies have shown a wide range of biological activities of different cyclitols, including *myo*-inositol, d-*chiro*-inositol, and d-pinitol. *myo*-Inositol treatment has been shown to prevent biochemical changes triggered by kainate-induced status epilepticus [11]. Qureshi and Al-Bedah reported that patients suffering from clinical depression, bulimia, epilepsy, panic disorder, unipolar and bipolar depression, and obsessive–compulsive disorder taking high-dose *myo*-inositol supplement showed promising results [12]. Among other important properties, *myo*-inositol possesses insulin-mimetic properties and is efficient in lowering postprandial blood glucose [13]. Moreover, *myo*-inositol and d-*chiro*-inositol improve insulin resistance in obese women and women with gestational diabetes [14], in addition to the activities of *myo*-inositol and *scyllo*-inositol [15]. A 3-*O*-methyl-d-*chiro*-inositol called d-pinitol also has substantial pharmacological importance [13]; it has been widely used in treatment of hypertension, rheumatism, and cardiovascular diseases, as an anticonvulsant, and for acquired immunodeficiency syndrome (AIDS) and neurological disorders [16–18]. Therefore, inositols as pharmaceutical ingredients are in great demand worldwide.

Phytoestrogens are a class of naturally occurring plant nutrients that have the ability to cause estrogen-like effects on the body (sexual hormones) due to their structural similarity to estradiol (17-β-estradiol), the primary female sex hormone [19]. Phytoestrogens belong to a large group of substituted natural phenolic compounds known as flavonoids. Several groups of flavonoids have estrogenic properties. Among these, coumestans, prenylflavonoids, and isoflavones possess the greatest estrogenic activity [20, 21]. Several studies have reported health benefits of phytoestrogens including lowered risk of osteoporosis, carcinogenesis, atherosclerosis, brain function disorders, obesity, and menopausal symptoms [22–24]. Moreover, phytoestrogens play an important role in plant defense systems, mainly against fungi [25], and control female fertility to prevent overpopulation and fertility of animals that may eat them to reduce further attacks [26].

Other important bioactive compounds are oligosaccharides. Lopez et al. [27] reported that oligosaccharides improve metabolic absorption of certain minerals even when phytate is present.

Isolation, separation, and analysis of these compounds are of great interest in several fields, such as pharmaceutical and food industries, biochemistry, medical cell biology, and biotechnology research or nutrition [11, 28–34]. The cost of their synthesis can be considerably higher than their isolation from plants [35, 36], which are rich sources of these compounds.

The approaches to isolate and concentrate these compounds are determined by the chemical nature of the matrix, the properties of the individual component or the sum of their parts, and the method of analysis. Sample preconcentration, sample cleaning, and separation of cyclic polyols from other chemical constituents are complicated due to their high polarity. The hydrophobicity value (expressed as log *P*) of *myo*-inositol is −2.11 (Table 1). Moreover, naturally occurring flavonoids in plant tissues can also play an interesting role, making it important to consider their presence in matrices. The hydrophobicity values of different classes of flavonoids are higher than for cyclic polyols and vary in the range from about 0 to 4 (Table 1). Prior to chromatographic analysis, different solvents have been used for extraction of cyclic polyols from plants, including water [37], ethyl acetate and methanol [38], and ethyl acetate and ethanol [39].

**Table 1:** Structures of some important cyclic polyols and flavonoids in plants

Structure	Common name/IUPAC name/class of chemicals	log P*
	*myo*-Inositol/(1*R*,2*R*,3*S*,4*S*,5*R*,6*S*)-cyclohexane-1,2,3,4,5,6-hexol/polyols	−2.11 ± 0.49
	Myricetin [62]/3,5,7-trihydroxy-2-(3,4,5-trihydroxyphenyl)chromen-4-one/flavonols	2.11 ± 0.74
	Luteolin [63]/2-(3,4-dihydroxyphenyl)-5,7-dihydroxychromen-4-one/flavones	2.40 ± 0.65
	Medicarpin [64]/(6*aR*,11*aR*)-9-methoxy-6*a*,11*a*-dihydro-6*H*-[1]benzofuro[3,2-*c*]chromen-3-ol/isoflavonoids	2.72 ± 0.36
	Pinocembrin [63]/(2*S*)-5,7-dihydroxy-2-phenyl-2,3-dihydrochromen-4-one/flavanones	3.93 ± 0.38
	Pelargonidin [65]/2-(4-hydroxyphenyl)chromenylium-3,5,7-triol/anthocyanidins	–
	Gallocatechin [66]/(2*R*,3*S*)-2-(3,4,5-trihydroxyphenyl)-3,4-dihydro-2*H*-chromene-3,5,7-triol/flavanols	−0.10 ± 0.38

* Predicted value of octanol–water partition coefficient, *P*, a descriptor of the hydrophobicity (or lipophilicity) of a substance

Nowadays, nanotechnology has growing importance in the field of separation science. Several manufacturers now offer nanomaterial composites with different geometric structures coated on silica gel for use as SPE sorbents for preconcentration from and cleanup of various matrices. Supercritical fluid extraction (SFE) followed by selective concentration on modified SPE cartridges allows commercial SFE units to reach high concentration coefficients while conserving the samples.

This review is focused on introduction of extraction methods and new packing materials for SPE cartridges for use on laboratory scale for isolation and preconcentration of some bioactive compounds from plants and biological matrices. Current examples of adsorbents for isolation and preconcentration of components based on chemical interactions rely on nonselective H-bonding interactions between target analytes and surface groups. To date, some work has been done on nonporous graphitized carbon [40], silica modified with nonpolar groups [41], followed by chromatographic methods of analysis [40, 42]. These methods are too slow, typically requiring time of hours, with low selectivity that makes them useless in real applications. The aim of this review is to summarize and discuss available data on the most relevant methods for isolation, purification, preconcentration, and separation of some active compounds from plants and other biological matrices such as urine samples, sesame oil, and honey from various floral sources. These methods include conventional extraction methods, modern extraction methods, solid-phase extraction using carbon-, polymer-, and silica-based solid-phase extraction cartridges, and membrane extraction.

## Extraction of Cyclic Polyols and Flavonoids

Extraction of bioactive compounds from plant materials has been intensively reviewed [43, 44]. Present interest is due to increased awareness of preventative healthcare, which could be promoted through consumption of plant material extracts. The different techniques employed for extraction of polyols and flavonoids can be classified into conventional and modern extraction methods. Conventional extraction methods include maceration and Soxhlet extraction, while the modern methods include pressurized liquid extraction (PLE), microwave-assisted extraction (MAE), ultrasound-assisted extraction (UAE), and supercritical fluid extraction (SFE). Extraction of solute using conventional extraction methods depends on the solubility of the solute from the plant in the solvent. A large quantity of solvent is often required to extract the desired solute, and/or use of elevated temperature, mechanical stirring, or shaking. On the other hand, modern extraction techniques consume less solvent and energy, with shorter extraction time, while preventing pollution.

The importance of polyols and flavonoids due to their pharmaceutical properties has attracted attention from many researchers. Isolation of these compounds from natural sources such as plants using different extraction methods has been performed to obtain the maximum possible concentrations.

Maceration is a conventional method for extraction of bioactive compounds from plant material, involving soaking of plant material in a specific solvent for a period of time, usually from a couple of hours up to several days. Ethanol and methanol are common solvents used for extraction of polyols and flavonoids by this technique, while water is often used to extract highly polar compounds such as polyols [45]. Ethanol is often preferred, probably due to environmental concerns. Cheng et al. [46] reported extraction of eight flavonoids from *Gynostemma pentaphyllum* using ethanol as solvent, while Sharma et al. [47] employed this method for isolation of the polyol d-pinitol from *Argyrolobium roseum* plant.

Soxhlet extraction is another conventional technique frequently used to isolate polyols and flavonoids from plant materials. Commonly, ethanol, methanol, and acetonitrile are used as solvent. This process involves heating a solvent to boiling, then returning the condensed vapors to the original flask. The main disadvantages of Soxhlet extraction are the time and solvent consumption, as at least 1 h and up to 72 h can be required for extraction. Various flavonoids have been extracted from leaves and flowers of *Cassia angustifolia* by Soxhlet extraction using ethanol in 5 h [48]. Also, some phytochemical compounds including *myo*-inositol and flavonoids were identified using gas chromatography by applying the same extraction technique to leaves of *Tephrosia purpurea* [49].

Pressurized fluid extraction is a green technique for plant material sample preparation prior to chromatographic analysis, utilizing conventional solvents at controlled temperature (50–200 °C) and pressure (10 and 1500–2000 psi). In addition, it uses less solvent (15–45 mL), requires shorter extraction time (15–25 min), and can be automated. Therefore, this technique has been widely applied in the fields of environmental, food, and pharmaceutical research. It is also known as accelerated solvent extraction (ASE) or enhanced solvent extraction (ESE). The efficacy of PLE for extraction of inositols has been studied and compared with other extraction methods. Higher inositol concentration (5.7 mg g^−1^) was obtained from pine (*Pinus pinea* L.) nuts using accelerated solvent extraction (conditions: 50 °C, 18 min, three cycles of 1.5 mL water each, 10 MPa) compared with solid–liquid extraction (3.7 mg g^−1^) (conditions: room temperature, 2 h, two cycles of 5 mL water each) [50]. PLE is also an effective technique for extraction of flavonoids from plant material. Bergeron et al. [51] reported extraction of different flavonoids from *Scutellaria lateriflora* using PLE at pressure of 10 MPa and various temperatures (85, 100, 150, 170, and 190 °C) with extraction time of 30 min in three cycles of 10 min.

Microwave-assisted extraction utilizes microwave energy to facilitate diffusion of analytes from the plant sample into the solvent. Water in plant material is responsible for absorbing the microwave energy, leading to internal superheating and cell structure perturbation, thus facilitating diffusion of biologically active compounds from the plant matrix. MAE has been applied for determination of isoflavonoids in *Radix astragali* [52]. The results showed that MAE achieved the highest extraction efficiency compared with other extraction methods such as Soxhlet, reflux, and ultrasonic extraction. The application of microwave extraction for isolation of flavonoids has been thoroughly reviewed by Routray and Orsat [53]. Regarding extraction of polyols, Ruiz-Aceituno et al. [54] compared the efficiency of microwave-assisted extraction and pressurized liquid extraction for extraction of inositols from artichoke external bracts using water as solvent, reporting that the final optimized MAE method at 60 °C for 3 min with 0.3 g of sample allowed extraction of slightly higher concentrations of inositol than PLE at 75 °C for 26.7 min (11.6 mg g^−1^ dry sample versus 7.6 mg g^−1^ dry sample) [54].

Ultrasound-assisted extraction is another environmentally friendly technique for extraction of bioactive compounds such as polyols and flavonoids from plants. It offers the same advantages as PLE and MAE in terms of reduced time and solvent use and lower energy consumption. This process involves use of ultrasound at frequencies ranging from 20 to 100 MHz to create cavitation bubbles in the solvent that disrupt plant cell walls and increase the surface contact between solvents and samples and the permeability of cell walls. UAE has been employed for extraction of bioactive compounds, such as polyols, flavonoids, and others. Žlabur et al. [55] investigated the influence of conventional and ultrasound-assisted extraction (frequency, time, and temperature) on the content of bioactive compounds including flavonoids obtained from lemon balm and peppermint leaves [55]. Meanwhile, Tetik and Yüksel [56] used UAE for extraction of d-pinitol from carob bods and studied the influence of different parameters such as temperature, ultrasonic power, dilution rate (material:water ratio), and time. Those authors reported that the highest concentration of d-pinitol was 11.98 g L^−1^, obtained using temperature of 50 °C, ultrasonic power of 207 W, dilution rate of 1:4, and extraction time of 120 min [56].

Supercritical fluid extraction (SFE) is one of the leading approaches for extraction of organic compounds from solid and in some cases semisolid matrices [57, 58]. The supercritical state is achieved when the temperature and pressure of a fluid are raised above the critical point, exhibiting unique properties in terms of compressibility, density, and viscosity that differ from those of gases or liquids in normal state. Cháfer and Berna [59] applied SFE for isolation of d-pinitol from carob pods at pressure of 20 or 30 MPa, temperature of 40 or 60 °C, and CO_2_ flow rate from 0.5 to 2.5 mL min^−1^, using water to trap the extract. The maximum extraction yield of flavonoids of approximately 4.24 mg g^−1^ from *Maydis stigma* flowers using SFE was obtained using temperature of 50.88 °C, pressure of 41.80 MPa, cosolvent amount of 2.488 mL g^−1^, and extraction time of 120 min [59].

Isolation of polyols from crude extracts by partition chromatography using ion-exchange resins and thin-layer chromatography results in low yields and recoveries, as well as being very expensive [60].

The advantages offered by CO_2_ as a fluid include its low critical temperature/pressure, low cost, high purity, low toxicity and reactivity, and higher quality of the extract [61]. For extraction of polar compounds such as polyols, a modifier polar cosolvent such as methanol, ethanol, acetonitrile, acetone, water, ethyl ether or dichloromethane can be added to increase the solubility. Also, an increase in the pressure of the supercritical fluid increases the solvent dipole moment and thus its “polarity.” Undoubtedly, SFE is one of the promising techniques for extraction of polyols and flavonoids.

## Solid-Phase and Membrane Extraction as Purification and Separation Techniques

Besides cyclic polyols and flavonoids, plant crude extracts usually contain a mixture of different compounds such as chlorophyll, carbohydrates, sugars, fats, and others. Therefore, no universal extraction procedure is suitable for extraction of just polyols or flavonoids. Different strategies including liquid–liquid partitioning, column chromatography, membrane extraction, and solid-phase extraction (SPE) are commonly used to remove unwanted components and obtain concentrated fractions of targets before analysis.

### Membrane Extraction as Separation Method for Flavonoids

Membrane extraction is an obvious alternative to classical liquid–liquid extraction due to the small volumes of organic solvent and high ratio of target sample to extractant [67, 68]. The most common membrane materials are cellulose acetate [69], polyamide [70], and polysulfone [71, 72]. A novel polysulfone–Fe_3_O_4_ composite ultrafiltration membrane was prepared and used to purify flavonoids from *Ginkgo biloba* extract (GBE) by adjusting the external magnetic field [73]. It was found that the content of flavonoids was superior when extraction was performed in a magnetic field. Zhang et al. [74] used a molecularly imprinted membrane prepared using luteolin as template molecule and aminopropyltriethoxysilane for separation of flavonoids.

### Solid-Phase Extraction (SPE)

SPE is a good choice as cleanup procedure for crude plant extracts, since it is rapid, reproducible, economical, and sensitive, clean extracts are obtained, and different cartridges with different sorbents can be used. Carbon- and polymer-based solid-phase extraction cartridges have been developed for separation and purification of some bioactive compounds including polyols, flavonoids, and oligosaccharides.

#### Carbon-Based Solid-Phase Extraction Cartridges

Carbon-based adsorbents are characterized by their polydisperse particle structure. However, under specific conditions, e.g., chemical activation of biomass waste sawdust using KOH, adsorbents with a desired narrow pore size distribution (*D* = 0.77–0.91 nm), high surface area (*S*_BET_ = 2000–3100 m^2^ g^−1^), and large micropore volume (*V*_p_ = 1.11–1.68 cm^3^ g^−1^) can be obtained [75]. Most carbon adsorbents [76–78] possess a heterogeneous pore network formed of macropores with width exceeding about 50 nm, mesopores with width from 2 to 50 nm, and micropores with width not exceeding about 2 nm.

Granular activated carbons are typical carbon-based adsorbents. These are nongraphitic and nongraphitizable carbons with highly disordered microstructure, i.e., “a non-graphitic carbon which cannot be transformed into graphitic carbon by high-temperature treatment up to 3026.85 °C under atmospheric pressure or a lower pressure” [79]. The pore network of carbon-based adsorbents and the chemical nature of the carbon surface appear to be the criteria that determine their sorption properties. Spaces between crystals form micropores where aromatic rings render the surface hydrophobic. The structure of the carbon skeleton can be considered a mixture of graphite-like crystallites forming hexagonal nets resembling a honeycomb, where each ring is formed from six carbon atoms with delocalized π-electrons. The surface chemistry of activated carbons creates oxygen-containing functionalities, the most important of which are carboxyl, hydroxyl, ether, carbonyl, lactones, and quinine [80].

Application of activated carbon (charcoal) in sample preparation of cyclic polyols and similar compounds is discussed here. Carbon-based classical adsorbents for fractionation of OH-containing organic compounds are produced from natural precursors such as coal, wood or coconut shell after treatment with different reagents such as acids and hydroxides. Activated coal has high specific surface area (*S*_BET_ > 500–1000 m^2^ g^−1^). Carbon-based sorbents are used in the form of nonporous graphitized carbon for fractionation and purification of cyclic polyols by SPE [40]. Redmond and Packer [81] described the application of SPE of oligosaccharides from dilute solutions using graphitized carbon, demonstrating its potential for purification and fractionation of mixtures of sugars based on their size and/or ionic character.

The feasibility of extracting natural organic compounds (flavonoids) from plant tissue or seed oil using limited amounts of carbon-based adsorbents was described recently [82]. Multiwalled carbon nanotubes (MWCNTs) have been reported as solid-phase adsorbents to extract polyphenols from honey samples [83–85]. Novel magnetic carboxylated MWCNTs treated with nitric acid, hydrogen peroxide, sulfuric acid, or a mixture of potassium permanganate and sulfuric acid were shown to be powerful sorbents capable of extracting small amounts of organic compounds [82]. Typically, any surface modification of a carbon-based adsorbent may alter its surface chemical properties and absorption capacities. The strategies available to fabricate carboxyl-containing nanotubes (c-MWCNTs) for use with various analytes have recently been expanded [86]. Furthermore, magnetic Fe_3_O_4_ particles were selected for the magnetic core because of their rapid, cheap, and high-yield synthesis, as well as strong magnetic separation properties [82]. Xiao et al. [87] have developed MWCNTs@SiO_2_ nanoparticles for simultaneous extraction and determination of traces of flavonoids. Multi- and single-walled carbon nanotube screen-printed electrodes (MWCNTs–SPE and SWCNTs–SPE) were used as sensors for flavonoids in presence of biocatalytic laccases, i.e., multi-copper proteins that use molecular oxygen to oxidize various aromatic and nonaromatic compounds by a radical-catalyzed reaction mechanism [88].

Carbon fibers coated with different stationary phases, which can be liquid (polymer) or solid (sorbent), can be used to extract different types of analyte. The approach and details of these newly synthesized carbon fibers (E-HyCu) have not been clarified due to a pending patent application by Sugitate and Saka [89]. However, carbon fibers play the role of two adsorbents: one with hydrophobic (C18) and the other with hydrophilic (carbon/NH_2_) groups. The efficiency of these new carbon fibers for removal of fatty acids and quercetin (flavonoid) was almost 100%. Deka and Saikia [90] also studied the separation and purification of flavonoids using activated carbon. Meanwhile, Ribeiro et al. [91] investigated selective removal of flavonoids from citrus juice using different adsorbents including granulated activated carbon, activated diatomaceous earths, and synthetic neutral resins (Amberlite XAD-4, XAD-7, and XAD-16). Since removal of reducing sugars, pigments, and vitamin C may also occur, the eventual adsorption of these compounds was also investigated.

Nanocomposites consisting of graphene oxide and Fe_3_O_4_ nanoparticles have been used as magnetic sorbents for extraction of flavonoids from green tea, red wine, and urine samples [92]. A solid-phase extraction method for efficient analysis of flavonoids using poly(1-vinyl-3-hexylimidazolium bromide)-graphene oxide-grafted silica has also been established and described [93]. Graphene-encapsulated silica was used as adsorbent in matrix solid-phase dispersion extraction of polymethoxylated flavonoids from dried *Murraya paniculata* (L.) Jack leaves [94] with recoveries above 92.61%.

#### Polymer-Based Solid-Phase Extraction Cartridges

**Fig. 1:**
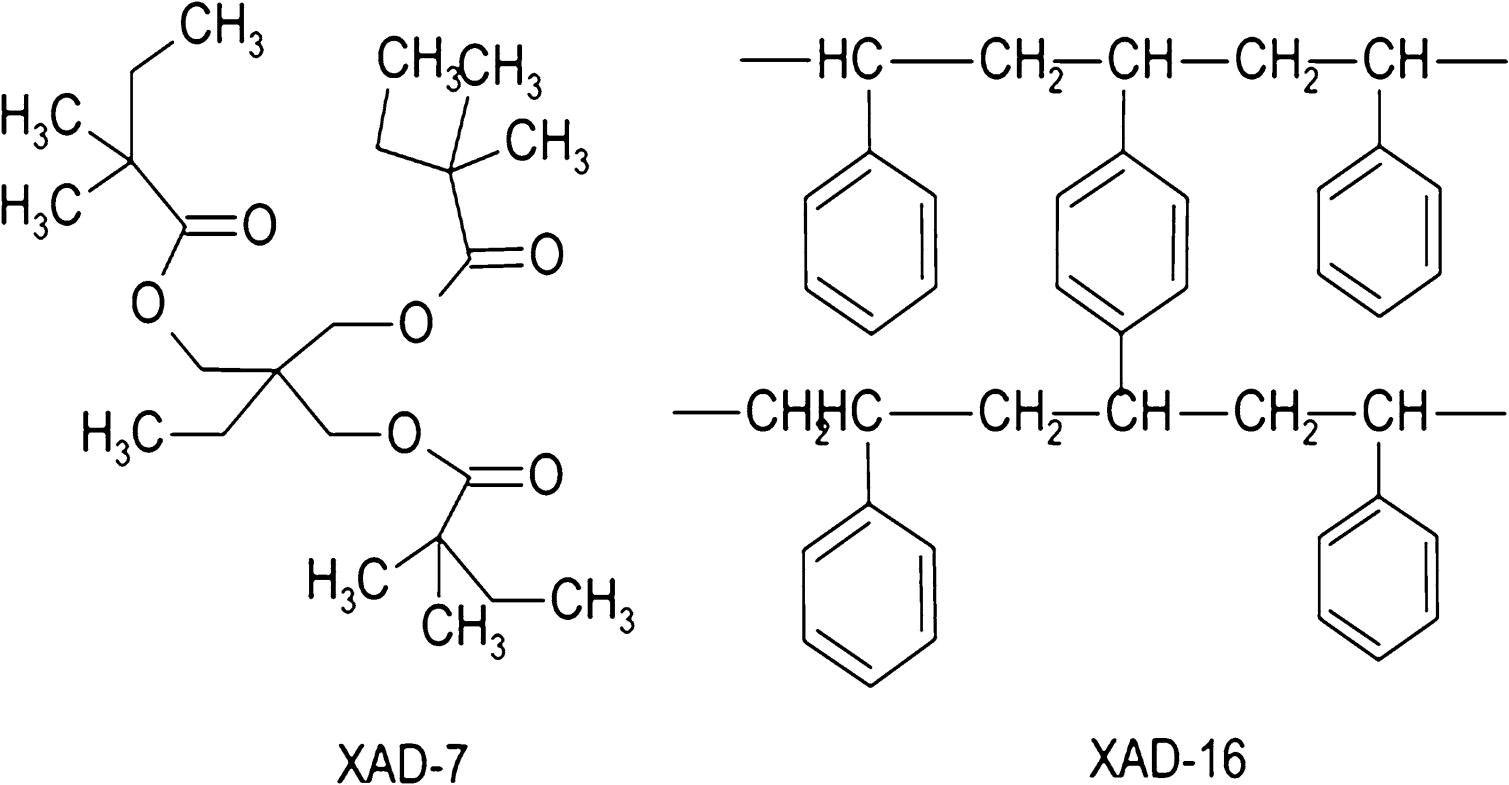
Structure of some polymer-based adsorbents

Polymer-based adsorbents are the most prominently used sorbents in SPE purification processes [95–97], due to their favorable stability and structural diversity, which allow modulation of their adsorption capacity and selectivity. Macroreticular polymeric resins have been widely used for adsorption, extraction, and purification of bioactive compounds from aqueous solutions and can be easily regenerated using appropriate elution solvent. Their adsorption of flavonoids can be ascribed to a synergy between hydrogen-bonding, electrostatic, π–π, and hydrophobic interactions of flavonoids with the sorbent. Synthetic neutral resins Amberlite XAD-4, XAD-7, and XAD-16 (Fig. 1) were tested for isolation of flavonoids from juice [91]. Amberlite XAD-4 and XAD-16 present similar structures, both being polystyrene-based resins, while XAD-7 is a polyacrylic-type resin. Amberlite XAD-7 has oxygen atoms in its structure and can form additional hydrogen bonds between two polar groups. Polyacrylic-type resin therefore might exhibit better absorption capacity towards polar compounds compared with Amberlite XAD-4 or XAD-16.

To address all the problems related to activated carbon, polymers with nonpolar and polar surfaces, particularly Amberlite XAD-4 based on styrene–divinylbenzene, cyano-functionalized porous polystyrene [98], biochars functionalized with polyethylimine, orthosilicate, and polyaniline [99], polyamide resins [100], aminophenyl boronic acid with uniform-macroporous poly(chloromethylstyrene-*co*-divinylbenzene), poly(chloromethylstyrene-*co*-divinylbenzene) [101], and polystyrene-divinyl sorbents with different degrees of crosslinking such as LiChrolut [102] or AMBERCHROM [103] were evaluated for solid-phase extraction of flavonoids from plant extracts.

**Fig. 2:**
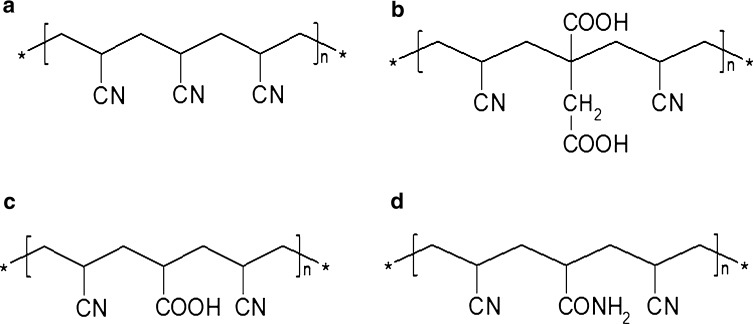
Chemical structure of synthesized polymers with different functional groups: polyacrylonitrile (**a**), poly(acrylonitrile-*co*-itaconic acid) (**b**), poly(acylonitrile-*co*-acrylic acid) (**c**), and poly(acrylonitrile-*co*-acrylamide) (**d**)

The hydrophobic surface of Amberlite XAD-4 results in weak contact between surface groups and hydrophobic groups of flavonoids, as well as low capacity for flavonoids compared with activated carbon [104, 105]. Analogs of Amberlite XAD-4 form donor–acceptor adducts with adsorbent molecules [106, 107]. Treatment of Amberlites with solvents (methanol, acetone, or acetonitrile) slightly increases their capacity but makes the SPE process more complex. Penner et al. [108] synthesized a new class of copolymers with higher capacity towards organic chemicals compared with copolymers such as the Amberlites. An example of such material is polysterene resin with high degree of crosslinking [108]. Its enhanced specific surface area (1500 m^2^ g^−1^) and bimodal porous structure resulted in increased recovery of organic compounds compared with polystyrene-divinylbenzene sorbents such as LiChrolut, ENVI-Chrom, or AMBERCHROM with 60% degree of crosslinking. Over the last few decades, chemically modified polymers have been applied for SPE, including β-cyclodextrin cross-linked polymer [109, 110] and different acrylic copolymers [67] containing CN–, –CONH_2_, and –COOH groups with different degrees of substitution, as presented in Fig. 2.

**Fig. 3:**
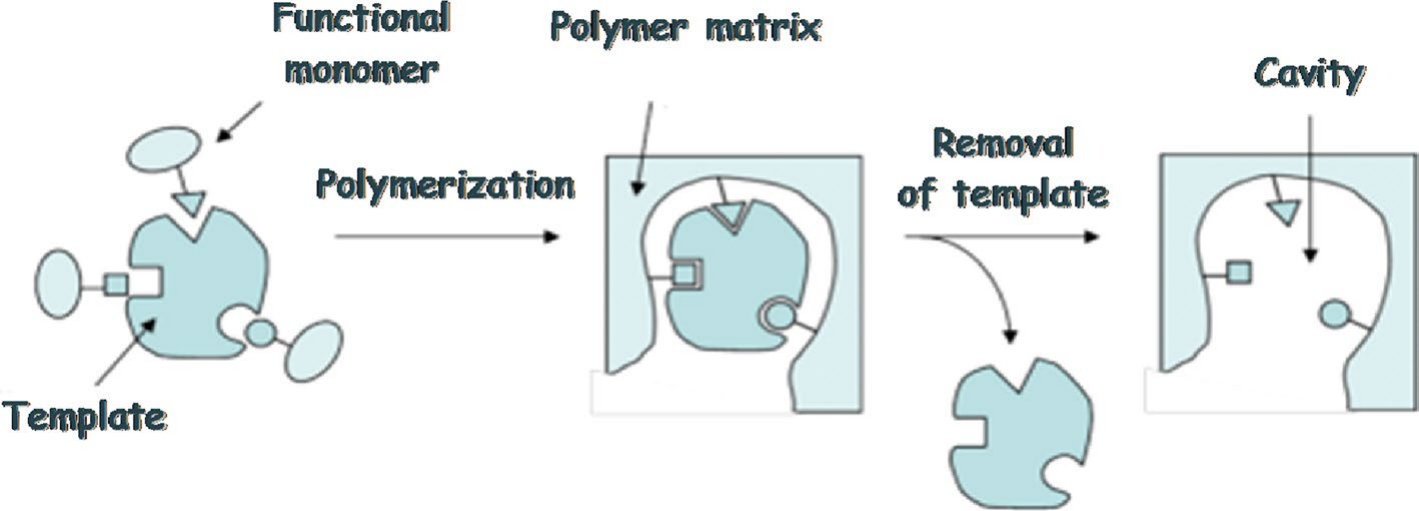
Scheme for molecular imprinting

This led to the introduction of smart adsorbents with a molecular fingerprint, called molecularly imprinted polymers (MIPs) in literature, offering significantly improved selective isolation [111, 112]. This approach is based on noncovalent imprinting, which involves formation of a host–guest complex via hydrogen bonds, ionic interactions, hydrophobic interactions, and metal ion interactions [113]. Figure 3 illustrates the synthesis process of MIPs, which mainly consists of three steps: (1) formation of a complex between the functional monomers and the template molecules through covalent or noncovalent interactions, (2) polymerization of the functional monomers in the presence of polymeric cross-linking reagent, and (3) removal of the template molecules from the polymer matrix to create three- dimensional cavities that are sterically and chemically complementary to the target analyte.

**Fig. 4:**
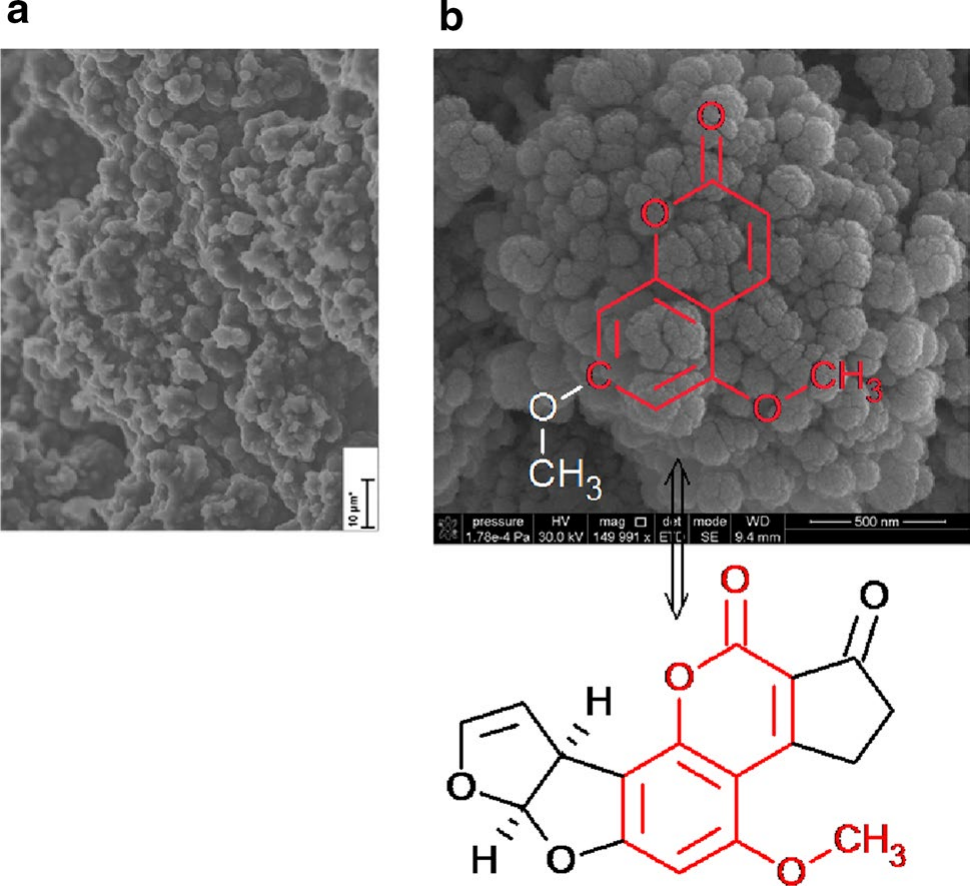
SEM images of polymer sorbents based on styrene–divinylbenzene: **a** Non-imprinted polymer (NIP) and **b** MIP with coumarin as template and aflatoxin B_1_ as analyte

The characteristics of the synthesized material surface can be investigated using classical physicochemical methods such as porosimetry, characterization of pore distribution, pore volume, pore diameter, and surface area, scanning electron microscopy (SEM) or transmission electron microscopy (TEM), atomic force microscopy (AFM), solid-state nuclear magnetic resonance (NMR), and Fourier-transform infrared (FTIR) spectroscopy to determine surface functional groups. The surface properties of thus-prepared sorbents and functional groups significantly influence their selectivity towards and recovery of target analytes (Fig. 4).

**Fig. 5:**
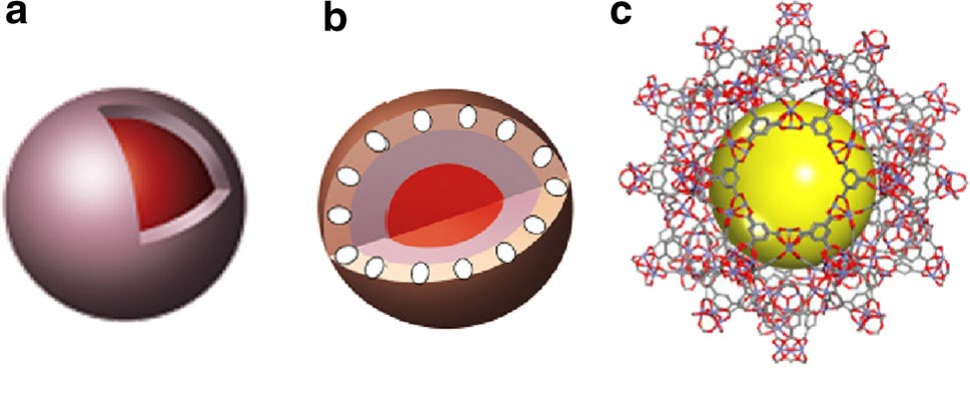
Type of adsorbent structure: **a** core–shell, **b** magnetic core–shell, and **c** metal–organic network (MOF)

Another new group of adsorbents based on MIP materials are so-called core–shell materials (Fig. 5a), whose sorption capacity and specificity can be controlled via their design, architecture, and physicochemical properties, for example, polymeric sorbents with nonporous center and porous outer layer as a mantle (Fig. 5b), in which magnetic Fe_3_O_4_ nanoparticles are localized. These materials, both polymeric and silica based, have been successfully used to purify the supernatant of complex biological samples (plant and animal tissues) [113].

Metal–organic frameworks (MOFs) are porous hybrid materials with a periodic three-dimensional network, possessing high porosity and specific surface area (Fig. 5c). Such materials are therefore characterized by high sorption capacity, which is important for sample preparation, especially in liquid–solid systems. Due to their merits, MOF materials have been widely used as candidates for molecular separation, gas storage, and catalysis [114]. The variety of applications and the ability for functionalization of the surface significantly influence the selectivity of the isolation process. Examples of such materials include polysaccharide-based sorbents such as cyclodextrins (CDs).

Cyclodextrins (CD) are cyclic oligosaccharides formed with *D*(+) glucopyranoside units bonded to each other through α(1 → 4) glycosidic bonds. Primary and secondary alcohol groups are located on the outer surface of the molecule. CDs have a hydrophobic inner core, while the outer surface of the cone is hydrophilic. Molecules of polar analytes form inclusion complexes with CDs through H-bonding. CD-based polymers are characterized by low specific surface area and the lack of a permeable porous network. This results in weaker interaction between the molecules of analytes and the grafted CD molecules.

Cartridges filled with HySphere GP (polymeric polydivinylbenzene phase) and HySphere SH (strong-hydrophobic modified polystyrene-divinylbenzene) have been proposed in literature [115, 116]. Although these polymers are selective towards flavonoids, their use can be time-consuming. However, they can decrease the limit of detection several fold due to the increased sample volume. Strong interactions between adsorbents and analytes result in lower limit of detection. Recovery of flavonoids using such adsorbents occurs as a result of nonspecific sorption by the matrix, formation of donor–acceptor adducts, or recovery due to an ion-exchange mechanism between surface-bound weak (or strongly basic) and weakly acidic groups of flavonoids.

**Fig. 6:**
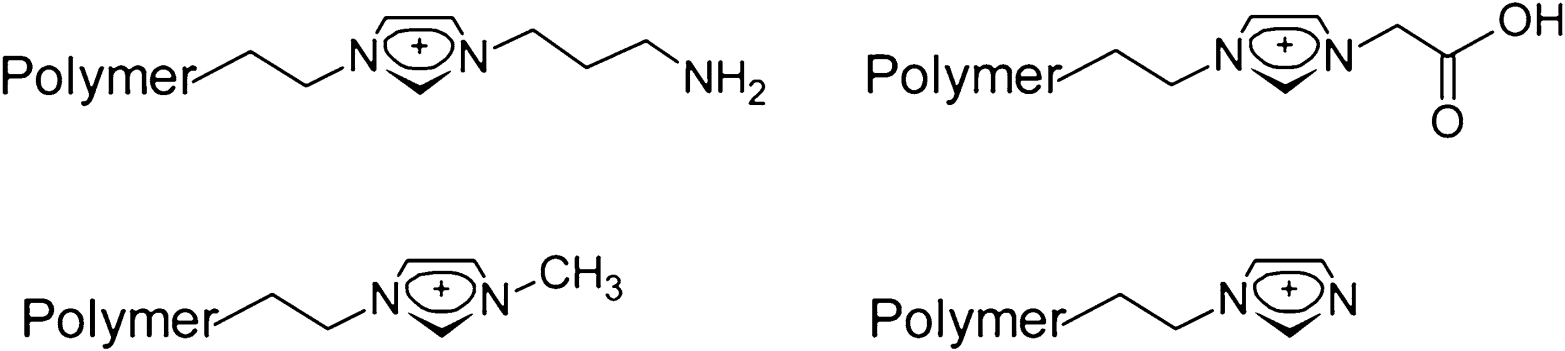
Chemical structure of imidazole-containing polymers

The aromatic rings and carbonyl groups of Amberlites behave as Lewis bases, while flavonoids behave as Lewis acids. Therefore, recovery of flavonoids will be easiest from media where they exist in molecular form. In recent years, polystyrene-divinylbenzene-based copolymers containing polar groups such as carbonyl, boronic acid, sulfonic acid, and nitrile groups have also been developed, where recovery occurs as a result of π-interactions or formation of hydrogen bonds. Imidazole polymers, methylimidazole polymers, carboxyl-imidazole polymers, and amino-imidazole polymers have also been employed for separation of polysaccharides [117] (Fig. 6). Polymers with a layer of copper phthalocyanine on polymer sorbent POLYSORB-2 represent additional examples of applications of polymers for SPE of antioxidants [118].

Chelation affects the formation of ionic bonds, resulting in higher affinity of the functional polymer towards diol-containing compounds. Both modified and unmodified organic polymers have been used to preconcentrate different classes of organic compound, including phenolic compounds, aldehydes, ketones, carboxylic acids, esters, and ethers [97, 119]. All the discussed polymer-based materials contain hydrophobic moieties (alkyl chains and aromatic groups) as well as hydrophilic groups. The hydrophilic fragments can be divided into neutral hydrophilic groups (cyano, amide, carbonyl), ionic groups (carboxyl, amino, sulfonic, imidazole ring), and zwitterionic groups (aminophenyl boronic acid). The complexity of ion-exchange sites (hydrophilic and hydrophobic moieties) leads to a lack of selectivity, poor recovery kinetics, and the need to use organic solvents, forcing researchers to seek more effective sorbents for SPE.

#### Other SPE Sorbents

In addition to the solvent and the pH stability of carbon and/or polymeric adsorbents in the range of 1–14, silica materials are still in use, mainly for nonpolar or medium-polar analytes. Silica-based sorbents are modified with groups such as C1, C2, C8, C18, CH, Ph or CN. One of the disadvantages of the most popular silica-based materials is the presence of residual silanol groups. When the water–organic mixture comes into contact with the stationary phase, organic solvent molecules are adsorbed onto the bonded functional groups via hydrophobic interactions, while water can adsorb on residual silanols via hydrogen bonding [113]. Moreover, presence of residual silanols can negatively influence the separation of polar analytes, especially basic compounds or biopolymers [113, 120, 121]. Additionally, to obtain more efficient recovery and a totally polar sorbent, the trends are to minimize the number of residual groups on the original silica. Furthermore, a trifunctional silane has been used for bonding *n*-alkyl chains with end-capping performed via trimethylsilane after bonding [122].

Stationary phases for adsorption include unmodified sorbents such as pure/bare silica, alumina, magnesium silicate, and diatomaceous earth. Their hydrogen-binding sites are mainly destroyed by water, which results in reduced retention of compounds of interest and poor reproducibility. For this reason, the surface of silica is functionalized by attaching polar groups such as cyano, amine, amide, and diol, resulting in a normal-phase sorbent that can extract a polar analyte from a polar medium via hydrophilic interactions. Such materials are mainly applied in clinical fields for purification of non-polar extracts such as hexane [123].

**Fig. 7:**
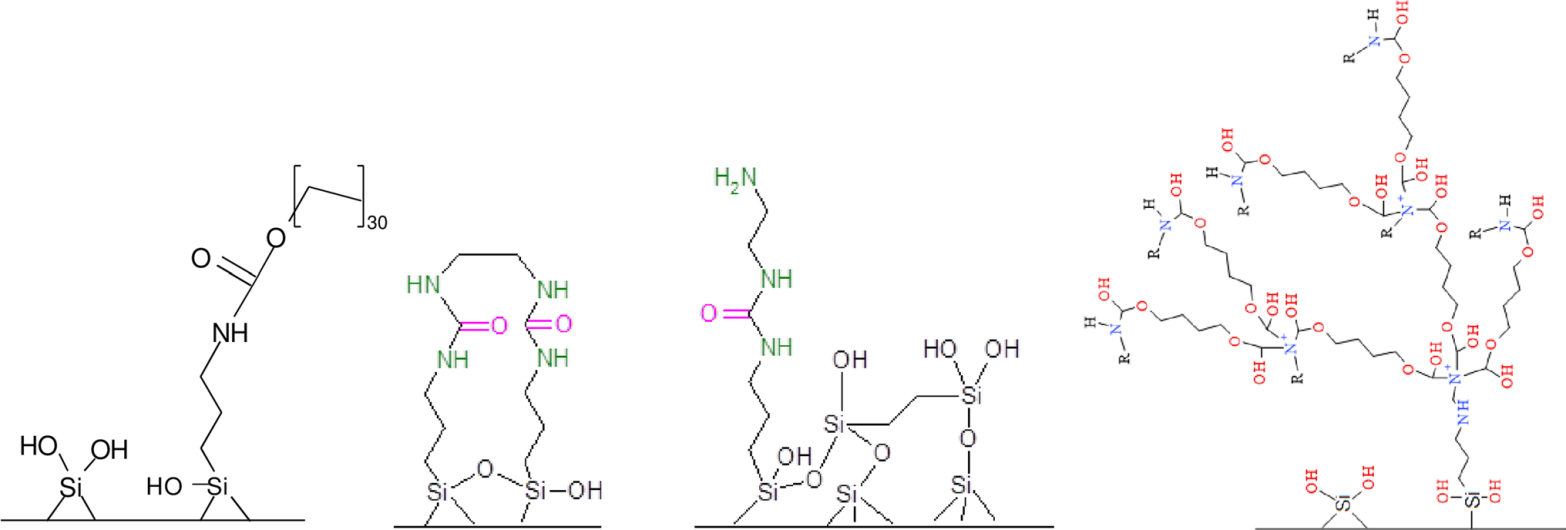
Structures of new sorbents for solid-phase extraction

Ion-exchange sorbents are characterized by ionic interactions, as in the case of negatively and positively charged analytes and biological fluids. Optimal conditions, including pH values, should be ensured. For this reason, depending on the nature of the analyte, use of new weak or strong cation- or anion-exchange stationary phases is recommended. Some of these contain specific active centers for selective isolation of analytes such as cations or anions. It is possible to control the polarity of both the stationary phase (surface shielding) and mobile phase (pH change) (Fig. 7).

The relationship between a target compound and its separation on the solid phase is mediated by hydrophobic interactions such as van der Waals forces as well as hydrophilic interactions such as dipole–dipole, induced dipole–dipole, hydrogen bonding, and π-interactions. Additionally, between the charged groups on the analyte of interest and the sorbent surface, electrostatic attractions as well as molecular recognition mechanisms are relevant. These types of interaction have been characterized for reversed-phase, normal-phase, ion-exchange, immunoaffinity, and molecularly imprinted polymers.

## Conclusions

This review has presented an overview of extraction techniques and separation methods for isolation of various biologically active compounds including cyclic polyols, flavonoids, and oligosaccharides from plant sources, as well as the advantages and disadvantages of each technique, and provided information about SPE as a method for separation and sample preparation before analysis. In addition, some commercial and synthetic SPE sorbents and their application for separation of selected components were presented. Conventional extraction methods were probably the first to be used for extraction of bioactive compounds present in plant materials using appropriate solvents. Compared with those methods, modern extraction techniques consume less solvent, hazardous chemicals, and energy, save time, and prevent pollution. SPE represents the best alternative compared with other purification methods for isolation of chemical constituents of plants. The long contact time during sorption of target analytes on activated carbons, as well as their universality towards different classes of organic compound, make them preferable for purification of extracts compared with other SPE sorbent materials. Polymers with a wide variety of functional groups, surface area, and porosity have become clear alternatives to activated carbons. The sorption properties of polymer-based adsorbents depend on their structure, the type of chemical bond between their chains, as well as their elementary structural units, molecular mass, and composition.

Synthesis of new materials to achieve optimal conditions for selective isolation of cyclic polyols and flavonoids still requires further research and development. Such materials could represent promising alternatives for selective isolation and separation of biologically active compounds from plants, including those with estrogenic properties.

## Abbreviations

CD: Cyclodextrin
MAE: Microwave-assisted extraction
MIP: Molecularly imprinted polymers
MOF: Metal–organic frameworks
MWCNTs: Multiwalled carbon nanotubes
c-MWCNTs: Carboxyl-containing multiwalled carbon nanotubes
PLE: Pressurized liquid extraction
SFE: Supercritical fluid extraction
SPE: Solid-phase extraction
